# A Novel Vaccine Approach for Chagas Disease Using Rare Adenovirus Serotype 48 Vectors

**DOI:** 10.3390/v8030078

**Published:** 2016-03-10

**Authors:** Anitra L. Farrow, Binghao J. Peng, Linlin Gu, Alexandre Krendelchtchikov, Qiana L. Matthews

**Affiliations:** 1 Division of Infectious Diseases, Department of Medicine, University of Alabama at Birmingham, Birmingham, AL 35294, USA; afarrow@uab.edu (A.L.F.); jbpb43@uab.edu (B.J.P.); akrend@uab.edu (A.K.); 2 Division of Pulmonary, Allergy and Critical Medicine, Department of Medicine, University of Alabama at Birmingham, Birmingham, AL 35294, USA; linlingu_yz@hotmail.com; 3 Center for AIDS Research, University of Alabama at Birmingham, Birmingham, AL 35294, USA

**Keywords:** adenovirus serotype 48, antigen capsid-incorporation, amastigote surface protein-2, gp83, Chagas’ disease, vaccine, *Trypanosoma cruzi*

## Abstract

Due to the increasing amount of people afflicted worldwide with Chagas disease and an increasing prevalence in the United States, there is a greater need to develop a safe and effective vaccine for this neglected disease. Adenovirus serotype 5 (Ad5) is the most common adenovirus vector used for gene therapy and vaccine approaches, but its efficacy is limited by preexisting vector immunity in humans resulting from natural infections. Therefore, we have employed rare serotype adenovirus 48 (Ad48) as an alternative choice for adenovirus/Chagas vaccine therapy. In this study, we modified Ad5 and Ad48 vectors to contain *T. cruzi*’s amastigote surface protein 2 (ASP-2) in the adenoviral early gene. We also modified Ad5 and Ad48 vectors to utilize the “Antigen Capsid-Incorporation” strategy by adding *T. cruzi* epitopes to protein IX (pIX). Mice that were immunized with the modified vectors were able to elicit *T. cruzi*-specific humoral and cellular responses. This study indicates that Ad48-modified vectors function comparable to or even premium to Ad5-modified vectors. This study provides novel data demonstrating that Ad48 can be used as a potential adenovirus vaccine vector against Chagas disease.

## 1. Introduction

Chagas disease (American trypanosomiasis) is one of the 17 neglected tropical diseases (NTDs) affecting the world today [1,2]. *Trypanosoma cruzi (T. cruzi)* is the intracellular parasite that causes Chagas disease (CD) [3]. *T. cruzi* is transmitted to the mammalian host at the site of a triatomine bug bite [4]. (CD) is an illness that once was only common in the Latin America region [4], now the disease is responsible for 10–50,000 deaths/year and infecting 12–20 million people worldwide [5]. Chagas disease has two clinical stages, acute and chronic stage [6]. The acute stage occurs after initial infection but often goes unobserved due to mild symptoms [7]. The acute stage can be deadly among children and immunocompromised adults [8]. There are pharmacological treatments for the acute stage however; the treatments are highly toxic [9]. Nifurtimox and Benznidazol are current treatments for this infection [9]. These anti-parasitic drugs are 80% successful in curing the acute phase with severe side effects. When the acute stage is untreated, the disease becomes chronic [7], anti-parasitic drugs are ineffective in curing the chronic phase.

The chronic stage can remain asymptomatic for many years. There is no treatment for the chronic stage. One-third of CD patients develop chronic chagasic cardiomyopathy (CCC) associated with parasite persistence and immunological unbalance [10]. The most severe and frequent manifestation of CD is CCC, which is associated with inflammation, mycytolysis, and fibrosis and affects 20%–40% of infected individuals at 10–30 years after infection [4]. Currently, the therapeutic management only lessens CCC symptoms. CCC is characterized by heart fibrosis leading to a variety of pathophysiological problems such as diastolic and systolic dysfunction, increased risk of arrhythmias, sudden cardiac death and worsening heart failure. Currently, there is no vaccine available for the prevention or treatment of chronic CD. A vaccine is imperative for the millions of people at risk.

T cells and B cells have been shown to play critical roles in protection against *T. cruzi* infection. CD8^+^ T cells are important in both primary [11] and secondary [12,13] protective *T. cruzi* immunity. Both CD8^+^ and CD4^+^ lymphocytes have a significant role in control of disease progression related to Chagas disease. Specifically, activation of these cell types leading to the production of various cytokines such as, interferon gamma (INFγ) [14,15] and tumor necrosis factor alpha (TNFα) leads to host protection. As mentioned previously, this is a complex immune response that controls parasite persistence and disease progression. In this regard interleukins (IL) 4 and 10 are involved in control of myocarditis when expressed at appropriate levels [16]. In addition, IL-12 and 17 have been observed to control parasitemia and parasitemia induced myocarditis [17,18,19,20]. Furthermore, B-cell response is involved in not only the prevention of the initial parasite infection but control as well.

Adenoviruses (Ads) have been utilized as vaccine delivery vehicles for many different pathogens primarily due to their ability to induce strong immune responses. Adenovirus vectored vaccines are ideal candidates for inducing robust antigen-specific T-cell responses. Our group has engineered adenovirus serotype 5 (Ad5) as an antigen delivery system essentially because of its safety and capability to infect a variety of cell types. Unfortunately, the major disadvantage of utilizing Ad5 is the pre-existing immunity (PEI) to Ad5 vectors. It is estimated that 50% to 90% of the adult population has PEI due to the common cold [21,22,23,24]. One approach to circumvent PEI is to replace Ad5 with an Ad that is less seroprevalent. There are at least 65 known human Ad serotypes that are divided into groups based on sequence homology [25,26,27]. A strong adenovirus contender to replace Ad5 is Adenovirus serotype 48 (Ad48). Ad48 is less seroprevalent than Ad5 and it belongs to group D while Ad5 belongs to group C [28,29]. For this body of work a series of Ad48 vectors were modified for specific *T. cruzi* immunogenicity.

The worldwide prevalence of Ad48 (subgroup D) is unknown at this time. A seroprevalence study showed that Ad48 seroprevalence in the sub-Saharan African is low [29]. In addition, of note, the highest seroprevalence of human Ad infection in the Latin American country of Peru were from Ad subgroups C, B and E [30]. Similarly, the countries of Brazil, Cuba [31] and Mexico [32] also have a high seroprevalence of Ad subgroup C infection. In contrast, two other countries bordering Peru, Argentina [33] and Columbia [34,35], observed a predominance in Ad subgroup B. The speculation that Ad48 is in low seroprevalence in Latin American countries, where Chagas is prevalent makes it an ideal vector candidate.

We sought to determine if the Ad48-modified vectors could perform comparable or better than the Ad5-modified vectors. This body of work is novel not only based on using Ad48 in Chagas research, but also because of the utilization of the “Antigen Capsid-Incorporation” strategy in combination with Ad48 for the first time. The “Antigen Capsid-Incorporation” strategy is a genetic approach whereby antigen(s) of interest are directly displayed on the viral capsid surface. This strategy not only holds the potential to bypass Ad5 PEI through modifications of Ad5 neutralizing epitopes on capsid proteins [36], but also elicits robust immunity by directly presenting the antigens of interest to the host’s immune system, leading to the protection against infections [36,37,38,39].

Human Ad5 contains three major capsid proteins, hexon, fiber, and penton. In our previous study, the immunodominant region of *T. cruzi*’s trans-sialidase [40] was incorporated within the hypervariable region 1 (HVR1) of hexon (Ad5-gp83). Ad5-gp83 was able to significantly provide protective humoral immunity against *T. cruzi* infection when challenged with the Tulahuen strain of *T. cruzi* [15]. Ad5 contains five minor capsid proteins, VI, VIII, IX, IIIa, and IVa2. pIX is a cement protein as well as a transcriptional activator of Ad genes and it is able to reorganize host cell nuclear domains [41]. In addition, pIX demonstrates a capacity to tolerate significant genetic modifications, including the addition of large polypeptides [42,43,44,45] such as cell-type-specific targeting ligands [46,47,48] and antigens [49].

Based on the successful modification of Ad5 pIX, we speculated that pIX of Ad48 would serve as a suitable incorporation site for antigen incorporation. We reasoned that a vaccine vector including gp83 neutralizing epitope, amastigote surface protein-2 (ASP-2), or an epitope of the carboxyl region of amastigote surface protein-2 (ASP-C), respectively, would be beneficial for a *T. cruzi* vaccine vector. ASP-2 is a protein of yet unknown function in the biology of the parasite [50,51,52]; however, it has been important for pre-clinical *T. cruzi* vaccine efforts [53,54].

## 2. Methods

### 2.1. Cell Culture

Human embryonic kidney (HEK293) cells were obtained from and cultured in the medium recommended by the American Type Culture Collection (Manassas, VA, USA). The cell line was incubated at 37 °C and 5% CO_2_ under humidified conditions.

### 2.2. Generation of Recombinant Adenoviral Vectors

To generate Ad5-CMV-ASP-2, the DNA sequence corresponding to full length ASP-2 (GenBank: AY186572.1) plus His_6_ (GenScript, Piscataway, NJ, USA) was digested with restriction enzymes BglII and XbaI then ligated into BglII/XbaI-linearized pShuttle-CMV plasmid vector (Agilent, Santa Clara, CA, USA). The resulting plasmid was linearized by PmeI enzyme and then co-transformed with the adenoviral backbone plasmid pAdEasy-1 (Agilent) into competent *E. coli* BJ5183 bacteria (Stratagene, La Jolla, CA, USA). To create Ad5-pIX-ASP-C, a short DNA sequence which included the c-terminal immunodominant region of ASP-2 (AA 542-560) and NheI sticky ends was generated by annealing two oligonucleotides using the following oligonucleotides: (top/bottom strand sequences): 5′-CTAGCCAGTCGGGAGGATAACAGACAGTACAGCTTTGTGAACCACAGATTCACTCTTGTGTAG-3′ and 5′-CTAGCTACACAAGAGTGAATCTGTGGTTCACAAAGCTGTACTGTCTGTTATCCTCCCGACTGGGG-3′. The annealed product was ligated into a modified pShuttle vector that contained the DNA that encoded for Ad5 pIX gene and a NheI site at the c-term (pShlpIXNhe) [55]. The correct recombinant plasmid was linearized by PmeI, followed by homologous recombination with pAdEasy-1 vector in BJ5183 cells. To construct Ad5-pIX-gp83, an immunodominant epitope from *T. cruzi*’s glycoprotein 83 (gp83) [15] and NheI sticky ends were produced by annealing the following oligonucleotides: 5′-CTAGCCAAAATTTATTGGAAACAGCCAGTGGAAGGGACGAAGAGTTGGACGCTGTCGAAGCATCACCATCACCATCACTAG-3′ and 5′-CTAGCTAGTGATGGTGATGGTGATGCTTCGACAGCGTCCAACTCTTCGTCCCTTCCACTGGCTGTTTCCAATAAATTTTGG-3′. Ligation and homologous recombination were performed as described above.

To rescue the Ad5-modified vectors, the recombinant adenoviral genomes were digested with PacI, and transfected with PolyJet (SignaGen Laboratories, Ijamsville, MD, USA) into the HEK293 cells. Multi-step large-scale propagations of recombinant Ad5 vectors were performed after the vectors were rescued. Viruses were purified by double cesium chloride (CsCl) gradient ultracentrifugation and dialyzed against 10% glycerol in phosphate buffered saline (PBS) without Mg^2+^ or Ca^2+^ and 10% glycerol. Viruses were stored at −80 °C until use.

To generate Ad48-CMV-ASP-2, the full length ASP-2 was amplified by PCR using two primers that introduced a KpnI site on the 5′ end and an EcoRI site on the 3′ end of the ASP-2 fragment. The digested fragment was cloned into the multiple cloning site of the Ad48 adaptor plasmid, pAdApt48 [29]. To rescue the modified vector, the recombinant plasmid, along with pWE.Ad48.ΔE3.5orf6 [29] was co-transfected into HEK293 cells with PolyJet. The transfected cells were coated with agarose after 24 h and continued in culture at 37 °C in 5% CO_2_ until plaques formed. The plaques were then collected and processed for large-scale upscale. Viruses were purified by double CsCl gradient ultracentrifugation and dialyzed against 10% glycerol in PBS without Mg^2+^ or Ca^2+^ and 10% glycerol. Viruses were stored at −80°C until use. To generate Ad48-pIX-ASP-C, a short DNA sequence which included FLAG epitope, MfeI restriction site, and the c-terminal immunodominant region of ASP-2 (AA 542-560) was produced by annealing the following oligonucleotides: (top/bottom strand sequences): 5′-AATTGGATTATAAGGATGACGATGACAAGAGTCGGGAGGATAACAGACAGTACAGCTTTGTGAACCACAGATTCACTCTTGTGTAGC-3′ and 5′-AATTGCTACACAAGAGTGAATCTGTGGTTCACAAAGCTGTACTGTCTGTTATCCTCCCGACTCTTGTCATCGTCATCCTTATAATCC-3′. The annealed product was ligated into a modified pAdApt48 (Mfe1 was introduced to the adaptor plasmid). The recombinant vector was rescued, upscaled, and purified as previously described for Ad48-CMV-ASP-2. The same cloning procedure was used to create Ad48-pIX-gp83 vector. The following oligonucleotides were annealed: 5′-AATTGAAAATTTATTGGAAACAGCCAGTGGAAGGGACGAAGAGTTGGACGCTGTCGAAGCATCACCATCACCATCACTAGC-3′ and 5′-AATTGCTAGTGATGGTGATGGTGATGCTTCGACAGCGTCCAACTCTTCGTCCCTTCCACTGGCTGTTTCCAATAAATTTTC-3′. The annealed product was ligated into the modified pAdApt48. The recombination, rescue and purification steps were the same as described for Ad48-pIX-ASP-C. All incorporations were verified by DNA sequencing.

The physical titer of each purified vector was determined by measuring an absorbance at 260 nm and expressed as viral particles (VPs) per mL. The infectious particles (IPs) per mL were determined by tissue culture infectious dose (TCID_50_) assay [56].

### 2.3. Western Blot Analysis

Protein expression was analyzed from lysates of HEK293 cells infected with Ad5-CMV-ASP-2 and Ad48-CMV-ASP-2. The other modified vectors, Ad5-pIX-ASP-C, Ad5-pIX-gp83, Ad48-pIX-ASP-C, and Ad48-pIX-gp83 (5 × 10^9^ VPs/vector) were analyzed for the protein of interest incorporated within the capsid. The lysates and vectors were denatured by boiling and resolved on SDS-PAGE gels, followed by transfer onto polyvinylidene difluoride (PVDF) membranes, which were then blocked with 5% dry non-fat milk in PBST for 1 h. Thereafter, the membranes were incubated overnight at 4 °C with His_6_ MAb (1:5000 dilution in blocking buffer; GenScript), or FLAG-HRP antibody. After overnight incubation, the membranes were washed, and the His_6_ membrane was incubated with HRP-conjugated goat anti-mouse antibody (1:5000; Millipore, Temecula, MA, USA). The proteins were detected by using 3′3′-diaminobenzidine tablets (Sigma-Aldrich, St. Louis, MO, USA) [56].

#### 2.3.1. Whole Virus Enzyme-linked Immunosorbent Assay (ELISA) and Sera ELISA

In order to investigate the exposure-display of *T. cruzi* epitopes on the surface of the pIX modified vectors, whole virus ELISAs were performed. In brief, the ELISA plates were coated with serial dilutions of the Ad5-pIX-ASP-C, Ad5-pIX-gp83, Ad48-pIX-ASP-C, Ad48-pIX-gp83, or the controls (Ad5 and Ad48). The immobilized vectors were blocked on 96-well plates. The immobilized vector was incubated with His_6_ MAb (1:2000; GenScript) or FLAG-HRP antibody. The FLAG-incubated plate was washed and set up for the detection step. The His_6_-incubated plate was washed and incubated with the HRP-conjugated goat anti-mouse antibody (1:5000; Millipore). ELISAs were developed with the SIGMAFAST OPD peroxidase substrate (Sigma-Aldrich) and measured at OD 450 nm.

ELISA was performed to determine the ability of the pIX-modified vectors to induce humoral immune responses. Sera from the immunized mice were collected two weeks after prime and 2 weeks after boost. ELISA plates were coated with either 10 μM of gp83-18 peptide (KIYWKQPVEGTKSWTLSK) or ASP-C peptide (SREDNRQYSFVNHRFTLV) (GenScript) in 100 μL of 50 mM carbonate buffer per well as previously described in [16]. Unbound peptide was removed by washing with PBST buffer (1× PBS and 0.05% Tween 20). The plate was then blocked with 5% non-fat milk/PBST for 1 h at room temperature and 1 μL of serum samples (diluted 1:100) from control and immunized mice were applied to the plate and incubated for 2 h at room temperature. The plate was then extensively washed and blocked again followed by incubation with the HRP-conjugated goat anti-mouse antibody (1:5000; Millipore). ELISAs were developed with the SIGMAFAST OPD peroxidase substrate (Sigma-Aldrich) and measured at OD 450 nm. The amount of IgG in the sera was calculated based on a standard curve of mouse IgG protein.

#### 2.3.2. Mice Immunizations

Mice immunizations with vectors (Ad5-CMV-ASP-2, Ad5-pIX-ASP-C, Ad48-CMV-ASP-2, Ad48-pIX-ASP-C, Ad5-pIX-gp83, and Ad48-pIX-gp83) were performed to determine the *T. cruzi*-specific immunogenicity. Groups of seven *C57BL/6* mice (6–8 weeks old female) were immunized intramuscularly with the corresponding vectors (1 × 10^10^ or 3 × 10^10^ VP/mouse) at each time-point, with a two-week interval between prime, boost, and reboost. The animals were euthanized two weeks after reboost; subsequently spleens were collected for further analysis. The University of Alabama at Birmingham Institutional Animal Use and Care Committee approved the use of mice as described herein under the approved protocol number 141209997.

#### 2.3.3. Intracellular Cytokine Staining (ICCS) Assay

Splenocytes collected from immunized *C57BL/6* mice (four mice per group) were treated with ACK Lysing buffer (Life Technologies, Carlsbad, CA, USA) and the cell concentration was adjusted to 2 × 10^6^ cells/mL in 500 μL of cell culture medium containing CD107a-FITC (2 mg/mL), and Golgi stop (Monensin) (10 μg/mL), (BD Biosciences, San Jose, CA, USA). The ASP-C peptide, SREDNRQYSFVNHRFTLV (10 μM) was added to the experimental tubes; PMA (50 ng/mL) and ionomycin (1 μg/mL) (Sigma-Aldrich) were added to the positive control tubes. All tubes were incubated for 6 h at 37 °C in the presence of 5% CO_2_. The cells were washed with PBS+ 1% FBS and labeled with surface antibodies. The samples were stained with CD3-Pacific Blue hamster-anti-mouse (BD Biosciences), CD4-APC-efluor 780 anti-mouse (eBiosciences, San Diego, CA, USA), and CD8-PE rat-anti-mouse (BD Biosciences) to determine the surface phenotype. The cells were then washed twice with PBS+ 1% FBS and permeabilized with the Cytofix/cytoperm reagent (BD Biosciences) for 20 min at 4 °C in the dark. After being washed twice, cells were stained for intracellular markers TNFα-PE-Cy7 rat-anti-mouse and IFNγ-Alexa Fluor 700-rat-anti-mouse (BD Biosciences), for 40 min at 4 °C in the dark. Finally, cells were washed twice and fixed in 1% formalin. At least 100,000 CD3+ events were acquired from each sample using a Becton Dickinson LSR II flow cytometer (BD Biosciences) and data was analyzed using FlowJo Version 10 software (TreeStar). Lymphocytes were analyzed based on forward and side scatter profiles. The gates were set based on the media control and these gates were applied to all samples from the same individual for each time point. Cytokine production was measured from the CD3^+^CD4^+^ or the CD3^+^CD8^+^ gates relative to the media control values.

### 2.4. Statistical Analyses

Descriptive statistics, such as means and standard deviations, or standard error of the mean, were computed to study variables of interest. Statistical analyses were performed by the nonpaired two-tailed Student *t*-test, assuming equal variance. Statistical significance was defined as *p* < 0.05.

## 3. Results

### 3.1. Construction of Ad5 and Ad48 Vectors

**Figure 1:**
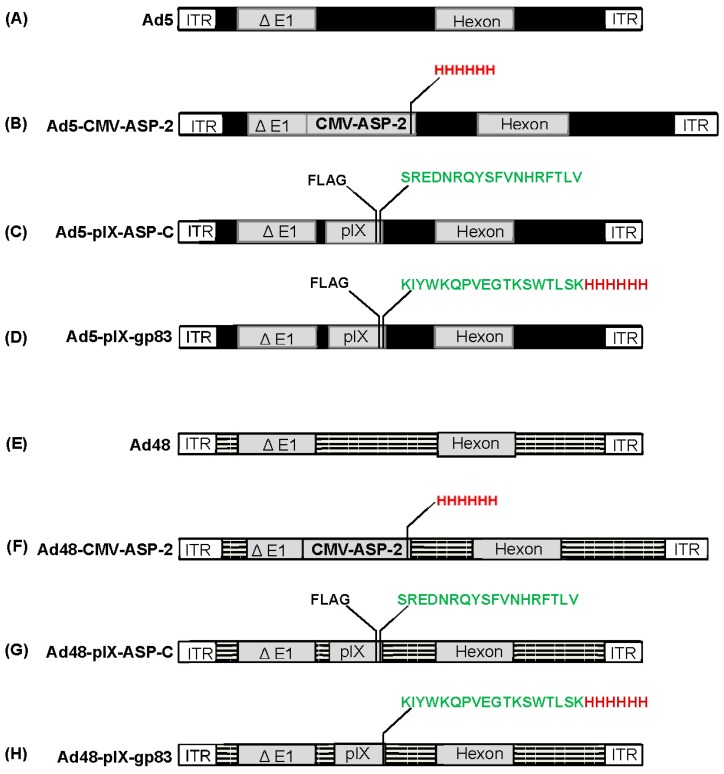
Schematic representation of the Ad5 and Ad48 modified vectors involved in this study. (**A**) Ad5, a replication-defective adenovirus with wildtype pIX; (**B**) Ad5-CMV-ASP-2, replication-defective Ad5 with full length ASP-2 plus His_6_ under the control of CMV promoter; (**C**) Ad5-pIX-ASP-C, Ad5 replication-defective genome containing an immunodominant region from the c-terminal of ASP-2 and a FLAG epitope within the pIX locale; (**D**) Ad5-gp83, Ad5 replication-defective genome containing an incorporated neutralizing *T. cruzi* trypomastigote gp83 epitope as well as His_6_ and FLAG epitopes within the pIX locale; (**E**) Ad48, a replication-defective adenovirus with wildtype pIX; (**F**) Ad48-CMV-ASP-2, replication-defective Ad48 with full length ASP-2 plus His_6_ under the control of CMV promoter; (**G**) Ad48-pIX-ASP-C, Ad48 replication-defective genome containing an immunodominant region from the c-terminal of ASP-2 and a FLAG epitope within the pIX locale; (**H**) Ad48-gp83, Ad48 replication-defective genome containing an incorporated neutralizing *T. cruzi* trypomastigote gp83 epitope within the pIX locale.

For this study, we have developed a series of recombinant Ad5 and Ad48 vectors, which are illustrated in Figure 1. We constructed Ad5 and Ad48 vectors expressing *T. cruzi*’s amastigote surface protein 2 (ASP-2) along with His_6_ under the transcriptional control of CMV (Figure 1B,F). We developed Ad5 and Ad48 vectors that have an immunodominant CD8 T-cell epitope (VNHRFTLV) from the carboxyl-terminal region of ASP-2 as well as a FLAG (DYKDDDDK) epitope incorporated into the minor Ad capsid protein IX (pIX) (Figure 1C,G). In addition, we also developed Ad5 and Ad48 vectors in which a *T. cruzi* gp83 trans-sialidase epitope [15] was incorporated into pIX (Figure 1D,H). A FLAG epitope was also incorporated onto pIX in the Ad5-pIX-modifed vector. The modified Ad genomes were partially sequenced to confirm that the correct genes were incorporated. Subsequent transfection of HEK293 cells with the sequenced verified recombinant Ad genomes resulted in rescue of the following vectors: Ad5, Ad5-CMV-ASP-2, Ad5-pIX-ASP-C, Ad5-pIX-gp83, Ad48, Ad48-CMV-ASP-2, Ad48-pIX-ASP-C, and Ad48-pIX-gp83.

### 3.2. Expression of ASP-2 and T. cruzi Epitopes

**Figure 2:**
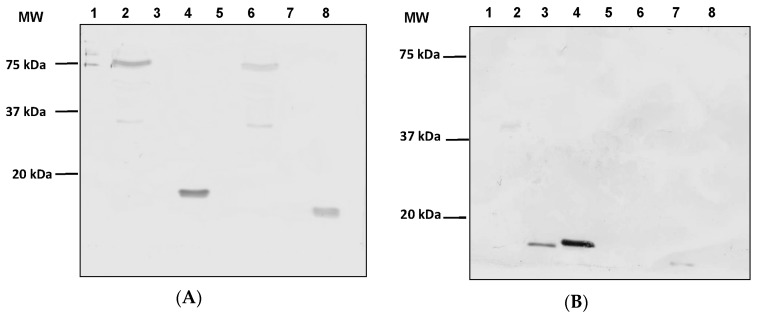
Verification of transgene expression and antigen capsid-incorporation of the modified vectors. (**A**) Western blot analysis confirmed the presence of His_6_ incorporation within the modified vectors. The proteins were detected with His_6_ MAb. Lane1, Ad5; lane 2, Ad5-CMV-ASP-2; lane 3, Ad5-pIX-ASP-C; lane 4, Ad5-pIX-gp83; lane 5, Ad48; lane 6, Ad48-CMV-ASP-2; lane 7, Ad48-pIX-ASP-C, lane 8, Ad48-pIX-gp83. (**B**) Western blot analysis confirmed the presence of FLAG incorporation within the modified vectors. Lane1, Ad5; lane 2, Ad5-CMV-ASP-2; lane 3, Ad5-pIX-ASP-C; lane 4, Ad5-pIX-gp83; lane 5, Ad48; lane 6, Ad48-CMV-ASP-2; lane 7, Ad48-pIX-ASP-C; lane 8, Ad48-pIX-gp83.

After successful incorporation of ASP-2 and *T. cruzi* epitopes, we next sought to verify expression of our transgenes and capsid incorporations at the protein level by Western blot analysis. Protein expression was analyzed from lysates generated from HEK293 cells infected with Ad5-CMV-ASP-2 and Ad48-CMV-ASP-2 as well as modified vectors Ad5-pIX-ASP-C, Ad5-pIX-gp83, Ad48-pIX-ASP-C, and Ad48-pIX-gp83. The lysates and vectors were denatured by boiling and resolved on SDS-PAGE gels. The probing of separated lysates and viral proteins of the modified vectors using His_6_ antibody detected the presence of protein bands with molecular weights of 78 kDa for Ad5-CMV-ASP-2 (Figure 2A, lane 2) and Ad48-CMV-ASP-2 (Figure 2A, lane 6), 19 kDa for Ad5-pIX-gp83 (Figure 2A, lane 4), and 18 kDa for Ad48-pIX-gp83 (Figure 2A, lane 8). As expected there was no protein band detected in lanes for Ad5-pIX-ASP-C (Figure 2A, lane 3) and Ad48-pIX-ASP-C (Figure 2A, lane 7) where His_6_ was not incorporated. There were also no protein band detected in the control lanes, Ad5 (Figure 2A, lane 1) and Ad48 (Figure 2A, lane 5). The probing of separated lysates and viral proteins of the modified vectors using FLAG antibody detected the presence of protein bands with molecular weights of 18 kDa for Ad5-pIX-ASP-C (Figure 2B, lane 3), 19 kDa for Ad5-pIX-gp83 (Figure 2B, lane 4), and 19 kDa for Ad48-pIX-ASP-C (Figure 2B, lane 7). As expected there was no protein band detected in lanes for the controls Ad5 (Figure 2B, lane 1) and Ad48 (Figure 2B, lane 5) as well as Ad5-CMV-ASP-2 (Figure 2B, lane 2), Ad48-CMV-ASP-2 (Figure 2B, lane 6), and Ad48-pIX-gp83 (Figure 2B, lane 8) where the FLAG epitope was not incorporated.

### 3.3. T. cruzi Antigens, Incorporated within pIX, Are Exposed on the Virion Surface

**Figure 3:**
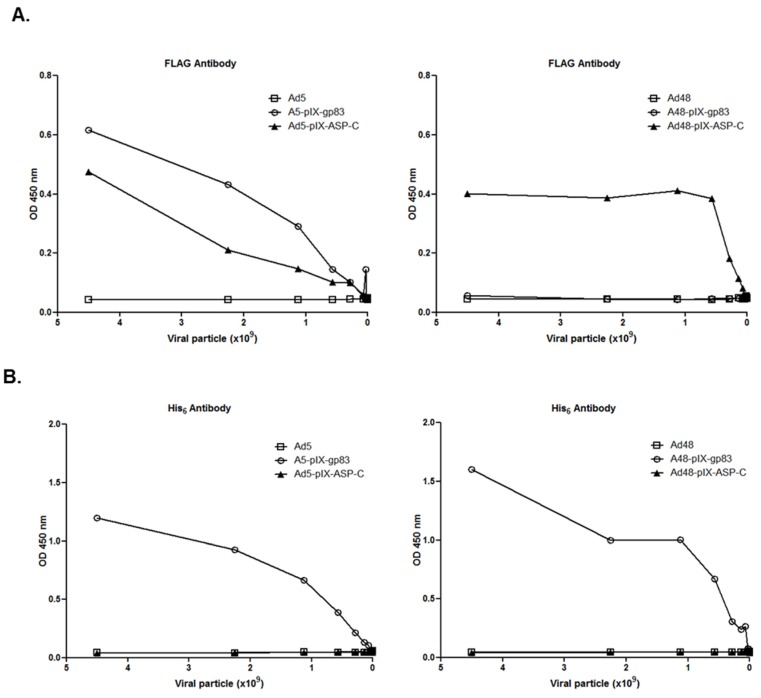
*T. cruzi* antigens are exposed on the virion surface. (**A**) Varying amounts (starting at 4.5 × 10^9^ VP/mL) of purified vectors were immobilized onto the wells of ELISA plates and incubated with FLAG-HRP antibody; (**B**) Varying amounts (starting at 4.5 × 10^9^ VP/mL) of purified vectors were immobilized onto the wells of ELISA plates and incubated with His_6_ MAb.

It is known that Ads can assemble and function with a defected/deleted pIX capsid protein [57]. For the modified pIX capsid incorporated vectors, we performed ELISA assays to verify that pIXs were intact and the *T. cruzi* antigens were accessible on the vectors’ surface (Figure 3). Serial diluted vectors were immobilized in the wells of an ELISA plate and incubated with either His_6_ or FLAG-HRP antibody. In reference to FLAG antibody, Ad5-pIX-ASP-C, Ad48-pIX-ASP-C, and Ad5-pIX-gp83 vectors showed substantial dose-dependent binding of FLAG antibody whereas no binding was seen in response to the controls, Ad5 or Ad48. There was also no binding with Ad48-pIX-gp83 as expected (Figure 3A). In reference to His_6_ antibody, there was substantial dose-dependent binding of His_6_ to Ad5-pIX-gp83, Ad48-pIX-ASP-C, and Ad48-pIX-gp83 whereas no binding was seen in response to the controls, Ad5 or Ad48 (Figure 3B). The data demonstrates that all modified pIX vectors assembled with functional pIX and the *T. cruzi* epitope incorporation was accessible.

### 3.4. Cellular Immune Response of Mice Immunized with Modified Ad5 and Ad48 Vectors

**Figure 4:**
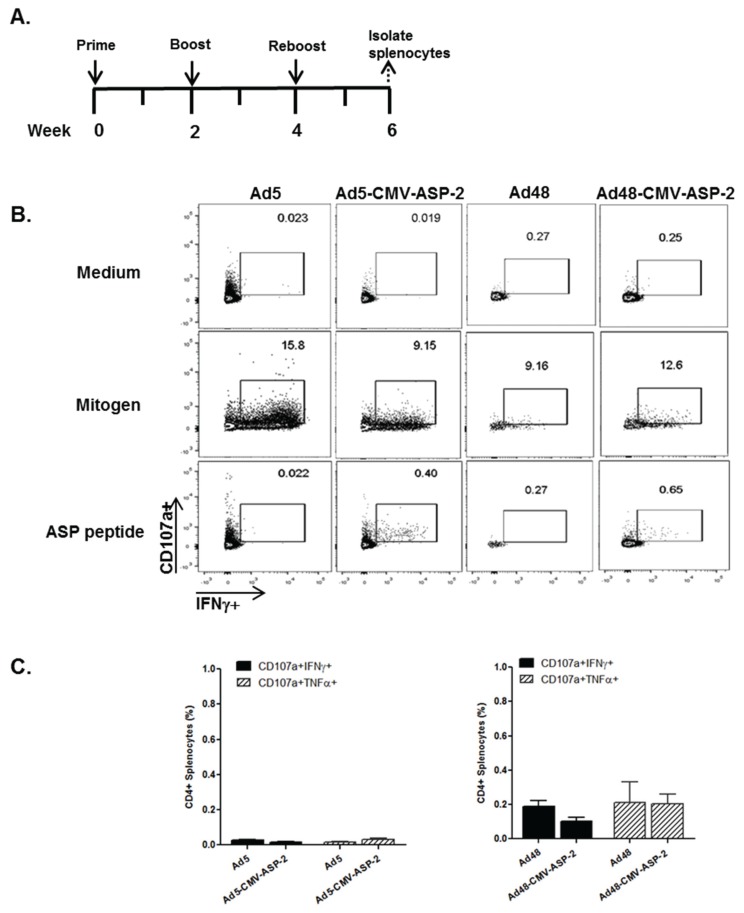

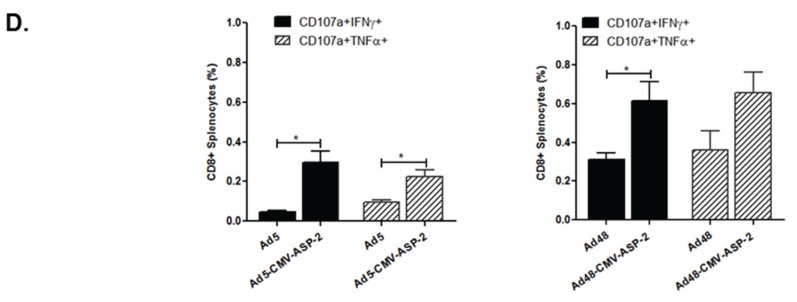
Frequencies of double positive phenotypes of ASP-specific T-cell responses in immunized mice. (**A**) Study design: *C57BL/6* mice were immunized intramuscularly with Ad5, Ad5-CMV-ASP-2, Ad48, or Ad48-CMV-ASP-2 at the indicated time points. Two weeks after the last immunization, splenocytes harvested from a subset of mice were stimulated with anti-CD107a and GolgiStop in the presence or absence of ASP-C peptide. Cells were gated by side scatter area (SSC-A); forward scatter area (FSC-A); and gated for CD3^+^ CD4^+^ or CD3^+^ CD8^+^ T lymphocytes; (**B**) Representative flow cytometry plots of CD107a^+^ and IFNγ^+^ by CD8^+^ T-cells from the spleens of the immunized mice; (**C**) Percentages of CD4^+^ T-cells which produce CD107a^+^IFNγ^+^ or CD107a^+^TNFα^+^ after stimulation with ASP-C peptide; (**D**) Percentages of CD8^+^ T-cells which produce CD107a^+^IFNγ^+^ or CD107a^+^TNFα^+^ after stimulation with ASP-C peptide. Results are presented as the mean ± SEM frequencies of CD4^+^ or CD8^+^ cells for 4 mice. (*) = *p* ≤ 0.05.

It is well documented that the CD8^+^ T-cell population plays an important role against resistance in parasitic infections [58,59,60], so it is imperative that modified Ad48 vectors elicit a strong cell-mediated immune response. To examine the T-cell profile generated by the Ad-modified vectors, *C57BL/6* mice were immunized intramuscularly with equal amounts (1 × 10^10^ VP/mL) of Ad5, Ad48, Ad5-CMV-ASP, or Ad48-CMV-ASP vectors according to the immunization schedule depicted in Figure 4A. Splenocytes from immunized mice were subjected to flow cytometry analysis. Splenocytes were stimulated with mitogen, gp83 peptide, or ASP-C peptide, gated for CD4^+^ and CD8^+^ phenotype, and analyzed for dual effector molecule secretion of T-cell degranulation marker, CD107a, and intracellular effector cytokines IFNγ and TNFα. Representative histograms of the double-positive gating for IFNγ+CD107a^+^ cells are shown in Figure 4B. There was no significant differences in the frequencies of peptide-specific CD4^+^ T-cells that mobilized CD107a to their surface and expressed IFNγ or TNFα compared to the control groups (Ad5 or Ad48) (Figure 4C). However, there was a significant difference with CD8^+^ T-cells. The Ad5-CMV-ASP-2 immunized mice showed a significant difference in the frequencies of peptide-specific CD8^+^ T-cells that mobilized CD107a to the cell surface and expressed IFNγ (*p* < 0.05) or TNFα (*p* < 0.05) compared to the Ad5 immunized mice (Figure 4D). We made a similar observation with the Ad48-CMV-ASP-2 immunized mice compared to Ad48 immunized mice. Figure 4D also indicated a significant difference in the frequencies of peptide-specific CD8^+^ T-cells that mobilized CD107a to the cell surface and expressed IFNγ (*p* < 0.05), but only an increasing trend in CD8^+^ T-cells that mobilized CD107a to the cell surface and expressed TNFα compared to the Ad48 immunized group.

**Figure 5:**
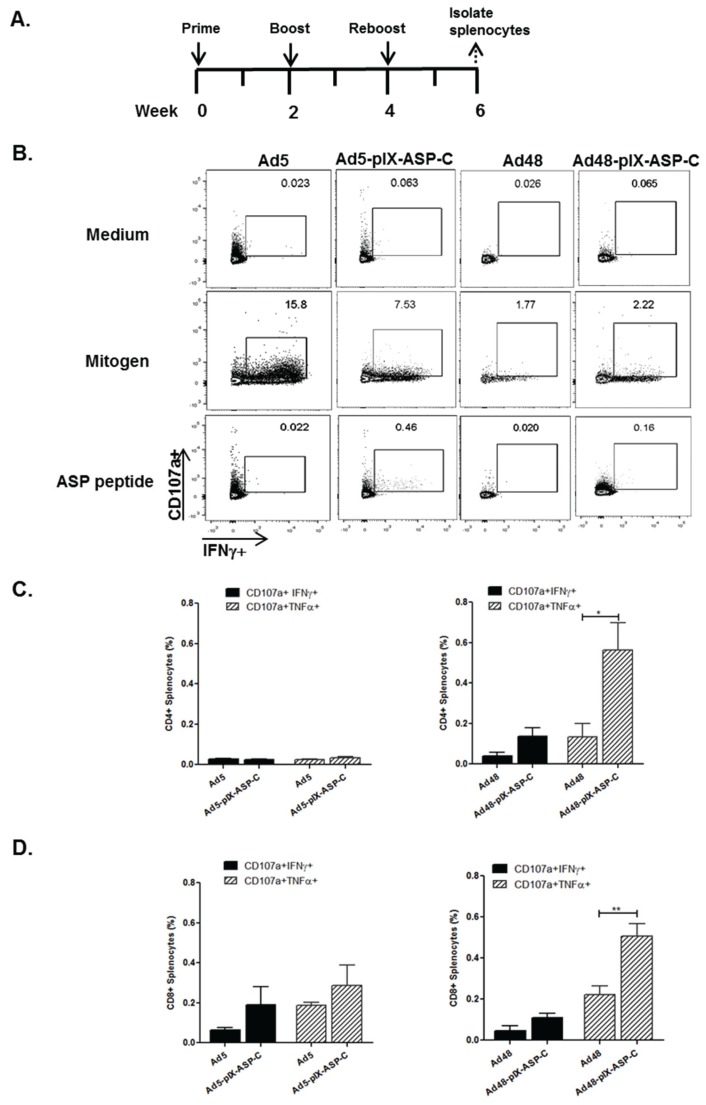
Frequencies of double positive phenotypes of *T. cruzi-*specific T-cell responses mice immunized with pIX-modified vectors. (**A**) Study design: *C57BL/6* mice were immunized intramuscularly with Ad5, Ad5-pIX-ASP-C, Ad48, or Ad48-pIX-ASP-C at the indicated time points. The experimental protocol was described in the legend of Figure 4; (**B**) Representative flow cytometry plots of CD107a and IFNγ by CD8^+^ T cells from the spleens of the immunized mice; (**C**) Percentages of CD4^+^ T-cells which produce CD107a^+^IFNγ^+^ or CD107a^+^TNFα^+^ after stimulation with ASP-C peptide; (**D**) Percentages of CD8^+^ T-cells which produce CD107a^+^IFNγ^+^or CD107a^+^TNFα^+^ after stimulation with ASP-C peptide. Results are presented as the mean ± SEM frequencies of CD4^+^ or CD8^+^ cells for 4 mice. (**) = *p* < 0.01, (*) = *p* ≤ 0.05.

We preformed identical experiments with the pIX-ASP-C vectors and followed the schedule depicted in Figure 5A. Representative histograms of the double-positive gating for IFNγ+CD107a^+^ cells are shown in Figure 5B. There were no differences in the percentages of CD4^+^+CD107a^+^IFNγ^+^ or CD4^+^+CD107a^+^+TNFα^+^ cells in Ad5-pIX-ASP-C immunized mice compared to the Ad5 immunized mice (Figure 5C). There was not a significant difference of CD4^+^+CD107a^+^IFNγ^+^ cells in Ad48-pIX-ASP-C immunized mice when compared to Ad48 immunized mice. Further analysis showed an increase of CD4^+^+CD107a^+^TNFα^+^ cells in Ad48-pIX-ASP-C immunized mice compared to Ad48 immunized mice (*p* < 0.05) (Figure 5C). Ad5-pIX-ASP-C immunized mice showed an increasing trend of the frequency of CD8^+^+CD107a^+^IFNγ^+^ and CD8^+^+CD107a^+^TNFα^+^ cells when compared to Ad5 immunized mice. Ad48-pIX-ASP-C immunized mice showed an increasing trend of the frequency of CD8^+^+CD107a^+^IFNγ^+^ when compared to Ad48 immunized mice. In addition, of important note, Ad48-pIX-ASP-C immunized mice showed a significant increase in the frequency of CD8^+^+CD107a^+^TNFα^+^ cells (*p* < 0.01) when compared to the Ad48 immunized mice (Figure 5D). Overall, all modified Ad vectors were able to improve the specific *T. cruzi* T-cell response when compare to the controls. When cross comparing the vectors, there was a significant increase in the frequency of CD8^+^+CD107a^+^IFNγ^+^ T-cells (*p* < 0.05) and CD8^+^+CD107a^+^TNFα^+^ T-cells (*p* < 0.05) from the Ad48-CMV-ASP-2 compared to Ad5-CMV-ASP-2. There was no significant T-cell response between Ad5-pIX-ASP-C and Ad48-pIX-ASP-C.

### 3.5. Humoral Immune Response to Capsid-Modified Vectors

**Figure 6:**
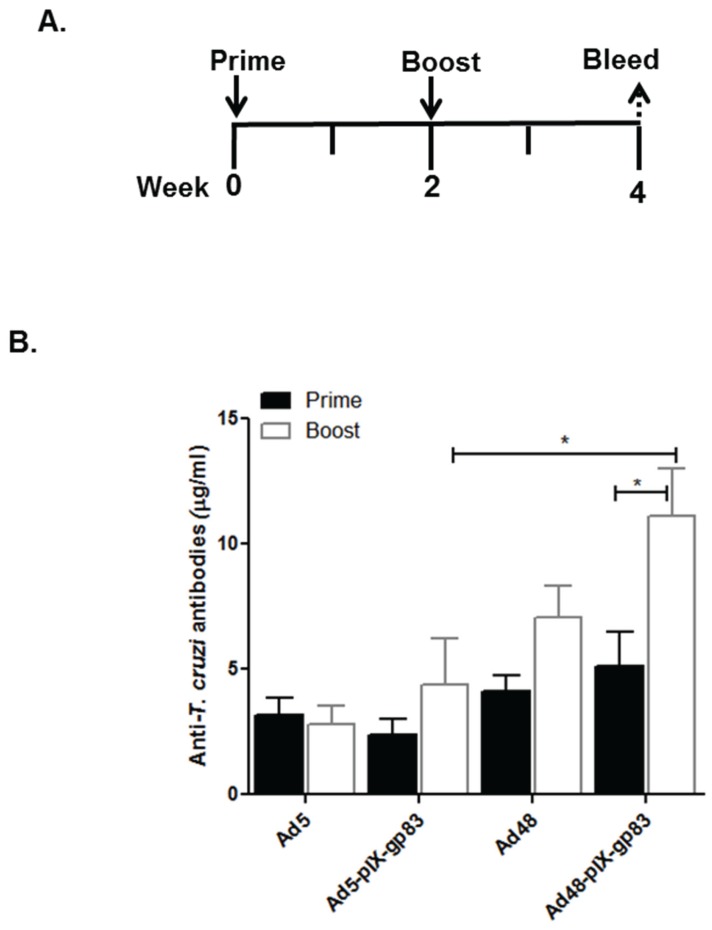

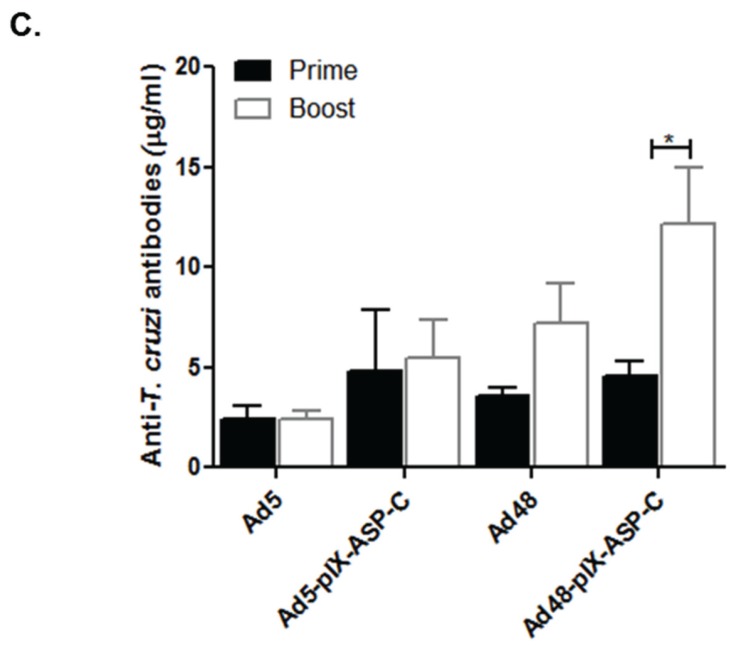
Antigen capsid-incorporation vector elicits an *in vivo* *T. cruzi* humoral immune response. (**A**) Study design: *C57BL/6* mice (*n* = 7) were primed and boosted with 3 × 10^10^ VP of Ad5, Ad5-pIX-gp83, Ad5-pIX-ASP-C, Ad48, Ad48-pIX-gp83, or Ad48-pIX-gp83. Two weeks after the last immunization serum was collected for ELISA binding assays. Either 10 μM of gp83 peptide or the ASP-C peptide was bound to ELISA plates; (**B**) Post-prime and post-boost serum from mice immunized with the *T. cruzi* gp83 modified vectors; (**C**) Post-prime and post-boost serum from mice immunized with the *T. cruzi* ASP-C pIX*-*modified vectors. The amount of anti-gp83 and anti-ASP-C antibodies in the sera was expressed as the mean ± SEM. (*) = *p* ≤ 0.05.

As stated in the introduction, pIX is an Ad minor capsid protein and has 1/3 the amount of copy numbers that the major capsid protein hexon contains [61]. For these set of experiments, the amount of vector used for immunization was increased from 1 × 10^10^ VP/mL to 3 × 10^10^ VP/mL. To determine the *T. cruzi*-specific antibody responses elicited by the gp83 vector, *C57BL/6* mice were immunized intramuscularly with 3 × 10^10^ VP/mL of Ad5, Ad48, Ad5-pIX-gp83, or Ad48-pIX-gp83 vectors according to the immunization schedule depicted in Figure 6A. Sera were collected from the immunized mice and evaluated for antibodies against *T. cruzi* by ELISA. The peptide specific for gp83, identical to what is in the capsid locale of the respective vectors was bound to ELISA plates. The plates were then incubated with the immunized mice sera. The binding was detected with HRP-conjugated secondary antibody. The total amount of IgG in sera was calculated based on a mouse IgG dilution standard curve. There was no significant difference of IgG levels between mice immunized with Ad5-pIX-gp83 and mice immunized with Ad5 (Figure 6B). There was also no significant difference of IgG levels between mice immunized with Ad48-pIX-gp83 and mice immunized with Ad48 (Figure 6B); however, there was a significant increase in the total IgG between prime and boost of the Ad48-pIX-gp83 immunized mice (*p* < 0.05) (Figure 6B). When cross-comparing the Ad5 and Ad48 groups, there was a significant increase of total IgG in the Ad48-pIX-gp83 immunized group compared to Ad5-pIX-gp83 immunized group (*p* < 0.05) (Figure 6B).

To determine if “Antigen Capsid-Incorporation” strategy would increase the humoral response to the T-cell specific Ad-modified vectors, *C57BL/6* mice were immunized intramuscularly with 3 × 10^10^ VP/mL of Ad5-pIX-ASP-C, Ad48-pIX-ASP-C, or the controls (Ad5 and Ad48) and followed the same protocol for ELISA as previously described. There was no significant difference between Ad5 immunized mice and Ad5-pIX-ASP-C immunized mice or between Ad48 immunized mice and Ad48-pIX-ASP-C immunized mice at prime or boost (Figure 6C). When comparing the IgG levels between prime and boost within the Ad5 groups, there was no significant increase (Figure 6C); however, there was a significant increase of anti-*T. cruzi* IgG levels detected at boost when comparing Ad48-pIX-ASP-C immunized mice to the IgG levels at prime (*p* < 0.05) (Figure 6C).

## 4. Discussion

We have developed novel Ad vectors that have the potential to optimize Ad vaccine approaches. In this manuscript we constructed Ad vectors for the development of a Chagas vaccine. We evaluated a series of Ad vectors of two serotypes, Ad5 and Ad48, whereby antigens were expressed (transgene antigen approach) or antigens were presented via the antigen capsid-incorporation strategy.

This manuscript highlights a two pronged approach: (1) the use of a rare serotype vector Ad48 in combination with the (2) Antigen Capsid-Incorporation strategy. The Ad48 vector was utilized because this serotype vector possesses many attributes that would make it an attractive alternative platform vector as compared to Ad5-based vector [62]. Some of these attribute included, namely its low seroprevalence in humans [63], failure to interact with factor X and transduce the liver [64,65], and its predictable cytokine profile, a reflection of its high-level accumulation in the spleen [62]. In this study, we utilized the antigen capsid-incorporation strategy, incorporating *T. cruzi* gp83 or *T. cruzi* ASP-C into the pIX locales of Ad5 and Ad48, respectively. To our knowledge this is the first time ever an antigen has been incorporated with the pIX locus of Ad48. *T. cruzi* antigen incorporation was validated at the genomic level in these vectors by sequencing and PCR analysis [66]. Antigen was confirmed at the protein level and within the viral capsid by western blot analysis (Figure 2) and ELISA analysis (Figure 3). This study herein, illustrated that the capsid incorporation of *T. cruzi* antigen is comparable between Ad5-modified vectors and Ad48-modified vectors (Figure 3). *T cruzi* antigens were also expressed within the deleted E1 region of Ad48 (Figure 2). To our knowledge this is the first time ever that Ad48 has been utilized for a Chagas vaccine vector. Expression of ASP-2 was also comparable between Ad5 and Ad48-modified vectors by means of protein quantitation compared to a standard.

Subsequently, these vectors were utilized in animal experiments to evaluate *T. cruzi*-specific humoral and T-cell responses. As shown in the data, we evaluated antigen-specific T-cell responses of conventional transgene expressing Ad5 and Ad48-modified vectors (Figure 4). Our data illustrate that Ad48-CMV-ASP-2 vector generated higher magnitude responses with respect to CD8^+^ CD107a^+^ (TNFα^+^ and IFNγ^+^) responses when compared to Ad5-CMV-ASP-2 vector (Figure 4D). In addition, we evaluated cell-mediated responses generated after immunization with antigen capsid-modified vectors (Ad5-pIX-ASP-C or Ad48-pIX-ASP-C) (Figure 5). After immunization with the Ad48-pIX-ASP-C vector, we observed significant *T. cruzi-*specific CD4^+^ T-cell and CD8^+^ T-cell responses ((*) = *p* ≤ 0.05, (**) = *p* < 0.01)). Whereas, immunization with Ad5-pIX-ASP-C did not show any *T. cruzi-*specific CD4^+^ and CD8^+^ responses (Figure 5C,D). To our knowledge this is the first demonstration that Ad48 has been utilized to generate a *T. cruzi-*specific response. Furthermore, this is the first demonstration of *T. cruzi-*specific responses generated from the Ad48 vector in combination with the Antigen Capsid-Incorporation strategy.

Studies were performed to determine *T. cruzi*-specific antibody responses elicited by the gp83 or ASP-C vectors (Figure 6). The data demonstrates that immunization with Ad48-pIX-gp83 elicits a superior and significant antibody response as compared to Ad5-pIX-gp83 at boost (*) = *p* ≤ 0.05 (Figure 6B). Of note, additional data demonstrates that immunizations with Ad48-pIX-ASP-C elicits a significant antibody response after boosting; whereas, homologous boosting of Ad5-pIX-ASP-C does not increase antibody specific response. It is an interesting phenomenon that Ad48-pIX-ASP-C elicits a significant antibody response after boosting because the ASP-C epitope incorporated within the vector is a CD8^+^ T-cell epitope and there are no known B-cell epitopes in the ASP-C epitope. This validates that the antigen capsid-incorporation strategy can induce a robust antigen-specific humoral immune response (Figure 6C). We speculate that the Ad48 vector provides better B cell help as compared to the blunted response observed in Figure 6C after priming and boosting with Ad5-pIX-ASP-C.

To our knowledge this is the first time ever an antigen has been incorporated within the pIX locus of Ad48. We have incorporated antigens within the pIX locale of Ad48 based on the plasticity of Ad5 pIX. In the future, we will explore the “Antigen Capsid-Incorporation” strategy in the context of additional Ad 48 minor (VI, VIII, IX, IIIa, and IVa2) and major (hexon, fiber, and penton) capsid proteins such as, hexon. Historically, the benefit of incorporating antigens within Ad5 hexon is that there is three times the amount of hexon monomers per Ad5 virion as compared to pIX. In theory, antigen incorporation within the hexon protein would yield a superior antigen-specific immune response as compared to pIX. However, incorporations within the pIX locale would be less detrimental to virion formation and stability as compared to antigen incorporation within hexon. As it relates to incorporations within the virion, pIX allows the incorporation of large epitopes/antigens [42,43,44,46,67], whereas hexon incorporation has a much smaller incorporation capacity [68]. However, a thorough examination of Ad48 hexon hypervariable regions will need to be assessed first to establish its utility for antigen incorporation.

## 5. Conclusions

Our data in this manuscript highlights a unique utility of rare serotype vector, Ad48. The Ad48 vector has not been utilized for *T. cruzi* immunizations prior to our study, herein. The focus of this manuscript is primarily to address vector-based questions, exploring the “Antigen Capsid-Incorporation” strategy within the context of the Ad48 vector system. Further studies are necessary to determine efficacy of a multivalent Ad48-based *T. cruzi* vaccine in challenge models, as well as immunogenicity in human populations.

## References

[1] Hotez P.J., Dumonteil E., Heffernan M.J., Bottazzi M.E. (2013). Innovation for the “bottom 100 million”: Eliminating neglected tropical diseases in the Americas. Adv. Exp. Med. Biol..

[2] Hotez P.J., Bottazzi M.E., Franco-Paredes C., Ault S.K., Periago M.R. (2008). The neglected tropical diseases of Latin America and the Caribbean: A review of disease burden and distribution and a roadmap for control and elimination. PLoS Negl. Trop. Dis..

[3] Chagas C. (1988). A short chronicle of the discovery of Chagas’ disease. Pacing Clin. Electrophysiol..

[4] Rassi A., Rassi A., Marin-Neto J.A. (2010). Chagas disease. Lancet.

[5] World Health Organization Chagas Disease (American trypanosomiasis). http://www.who.int/mediacentre/factsheets/fs340/en/.

[6] Biolo A., Ribeiro A.L., Clausell N. (2010). Chagas cardiomyopathy—Where do we stand after a hundred years?. Prog. Cardiovasc. Dis..

[7] Haberland A., Saravia S.G., Wallukat G., Ziebig R., Schimke I. (2013). Chronic Chagas disease: From basics to laboratory medicine. Clin. Chem. Lab. Med..

[8] Pinazo M.J., Espinosa G., Cortes-Lletget C., Posada Ede J., Aldasoro E., Oliveira I., Munoz J., Gallego M., Gascon J. (2013). Immunosuppression and Chagas disease: A management challenge. PLoS Negl. Trop. Dis..

[9] Castro J.A., Diaz de Toranzo E.G. (1988). Toxic effects of nifurtimox and benznidazole, two drugs used against american trypanosomiasis (Chagas’ disease). Biomed. Environ. Sci..

[10] Pereira I.R., Vilar-Pereira G., Marques V., da Silva A.A., Caetano B., Moreira O.C., Machado A.V., Bruna-Romero O., Rodrigues M.M., Gazzinelli R.T. (2015). A human type 5 adenovirus-based trypanosoma cruzi therapeutic vaccine re-programs immune response and reverses chronic cardiomyopathy. PLoS Pathog..

[11] Tarleton R.L., Koller B.H., Latour A., Postan M. (1992). Susceptibility of beta 2-microglobulin-deficient mice to *Trypanosoma cruzi* infection. Nature.

[12] Hoft D.F., Eickhoff C.S. (2002). Type 1 immunity provides optimal protection against both mucosal and systemic *Trypanosoma cruzi* challenges. Infect. Immun..

[13] Eickhoff C.S., Vasconcelos J.R., Sullivan N.L., Blazevic A., Bruna-Romero O., Rodrigues M.M., Hoft D.F. (2011). Co-administration of a plasmid DNA encoding IL-15 improves long-term protection of a genetic vaccine against *Trypanosoma cruzi*. PLoS Negl. Trop. Dis..

[14] Vasconcelos J.R., Bruna-Romero O., Araujo A.F., Dominguez M.R., Ersching J., de Alencar B.C., Machado A.V., Gazzinelli R.T., Bortoluci K.R., Amarante-Mendes G.P. (2012). Pathogen-induced proapoptotic phenotype and high CD95 (Fas) expression accompany a suboptimal CD8^+^ T-cell response: Reversal by adenoviral vaccine. PLoS Pathog..

[15] Farrow A.L., Rachakonda G., Gu L., Krendelchtchikova V., Nde P.N., Pratap S., Lima M.F., Villalta F., Matthews Q.L. (2014). Immunization with hexon modified adenoviral vectors integrated with gp83 epitope provides protection against *Trypanosoma cruzi* infection. PLoS Negl. Trop. Dis..

[16] Abrahamsohn I.A., da Silva A.P., Coffman R.L. (2000). Effects of interleukin-4 deprivation and treatment on resistance to trypanosoma cruzi. Infect. Immun..

[17] Aliberti J.C., Cardoso M.A., Martins G.A., Gazzinelli R.T., Vieira L.Q., Silva J.S. (1996). Interleukin-12 mediates resistance to *Trypanosoma cruzi* in mice and is produced by murine macrophages in response to live trypomastigotes. Infect. Immun..

[18] Abrahamsohn I.A., Coffman R.L. (1996). *Trypanosoma cruzi*: IL-10, TNF, IFN-γ, and IL-12 regulate innate and acquired immunity to infection. Exp. Parasitol..

[19] Da Matta Guedes P.M., Gutierrez F.R., Maia F.L., Milanezi C.M., Silva G.K., Pavanelli W.R., Silva J.S. (2010). IL-17 produced during *Trypanosoma cruzi* infection plays a central role in regulating parasite-induced myocarditis. PLoS Negl. Trop. Dis..

[20] Miyazaki Y., Hamano S., Wang S., Shimanoe Y., Iwakura Y., Yoshida H. (2010). IL-17 is necessary for host protection against acute-phase *Trypanosoma cruzi* infection. J. Immunol..

[21] Chirmule N., Propert K., Magosin S., Qian Y., Qian R., Wilson J. (1999). Immune responses to adenovirus and adeno-associated virus in humans. Gene Ther..

[22] Kass-Eisler A., Leinwand L., Gall J., Bloom B., Falck-Pedersen E. (1996). Circumventing the immune response to adenovirus-mediated gene therapy. Gene Ther..

[23] Wohlfart C. (1988). Neutralization of adenoviruses: Kinetics, stoichiometry, and mechanisms. J. Virol..

[24] Schagen F.H., Ossevoort M., Toes R.E., Hoeben R.C. (2004). Immune responses against adenoviral vectors and their transgene products: A review of strategies for evasion. Crit. Rev. Oncol. Hematol..

[25] Matsushima Y., Shimizu H., Kano A., Nakajima E., Ishimaru Y., Dey S.K., Watanabe Y., Adachi F., Suzuki K., Mitani K. (2012). Novel human adenovirus strain, bangladesh. Emerg. Infect. Dis..

[26] Geisbert T.W., Bailey M., Hensley L., Asiedu C., Geisbert J., Stanley D., Honko A., Johnson J., Mulangu S., Pau M.G. (2011). Recombinant adenovirus serotype 26 (Ad26) and Ad35 vaccine vectors bypass immunity to Ad5 and protect nonhuman primates against ebolavirus challenge. J. Virol..

[27] Coughlan L., Alba R., Parker A.L., Bradshaw A.C., McNeish I.A., Nicklin S.A., Baker A.H. (2010). Tropism-modification strategies for targeted gene delivery using adenoviral vectors. Viruses.

[28] Thorner A.R., Vogels R., Kaspers J., Weverling G.J., Holterman L., Lemckert A.A., Dilraj A., McNally L.M., Jeena P.M., Jepsen S. (2006). Age dependence of adenovirus-specific neutralizing antibody titers in individuals from sub-Saharan Africa. J. Clin. Microbiol..

[29] Abbink P., Lemckert A.A., Ewald B.A., Lynch D.M., Denholtz M., Smits S., Holterman L., Damen I., Vogels R., Thorner A.R. (2007). Comparative seroprevalence and immunogenicity of six rare serotype recombinant adenovirus vaccine vectors from subgroups B and D. J. Virol..

[30] Ampuero J.S., Ocana V., Gomez J., Gamero M.E., Garcia J., Halsey E.S., Laguna-Torres V.A. (2012). Adenovirus respiratory tract infections in Peru. PLoS ONE.

[31] Pumariega T., Savon C., Mune M., Cancio R., Gonzalez G., Valdivia A., Gonzalez Z., Goyenechea A. (2000). Isolation and identification of adenovirus in hospitalized children, under five years, with acute respiratory disease, in Havana, Cuba. Mem. Inst. Oswaldo Cruz.

[32] Rosete D.P., Manjarrez M.E., Barron B.L. (2008). Adenoviruses C in non-hospitalized Mexican children older than five years of age with acute respiratory infection. Mem. Inst. Oswaldo Cruz.

[33] Barrero P.R., Valinotto L.E., Tittarelli E., Mistchenko A.S. (2012). Molecular typing of adenoviruses in pediatric respiratory infections in Buenos Aires, Argentina (1999–2010). J. Clin. Virol..

[34] Herrera-Rodriguez D.H., de la Hoz F., Marino C., Ramirez E., Lopez J.D., Velez C. (2007). Adenovirus in children under five years of age. Circulation patterns and clinical and epidemiological characteristics in Colombia, 1997–2003. Revista de Salud Pública.

[35] Rojas L.J., Jaramillo C.A., Mojica M.F., Escalante M.P., Delgado P. (2012). Molecular typing of adenovirus circulating in a colombian paediatric population with acute respiratory infection. Epidemiol. Infect..

[36] Sharma A., Krause A., Xu Y., Sung B., Wu W., Worgall S. (2013). Adenovirus-based vaccine with epitopes incorporated in novel fiber sites to induce protective immunity against *Pseudomonas aeruginosa*. PLoS ONE.

[37] Palma C., Overstreet M.G., Guedon J.M., Hoiczyk E., Ward C., Karen K.A., Zavala F., Ketner G. (2011). Adenovirus particles that display the *Plasmodium falciparum* circumsporozoite protein NANP repeat induce sporozoite-neutralizing antibodies in mice. Vaccine.

[38] Worgall S., Krause A., Rivara M., Hee K.K., Vintayen E.V., Hackett N.R., Roelvink P.W., Bruder J.T., Wickham T.J., Kovesdi I. (2005). Protection against *P. aeruginosa* with an adenovirus vector containing an OprF epitope in the capsid. J. Clin. Investig..

[39] McConnell M.J., Danthinne X., Imperiale M.J. (2006). Characterization of a permissive epitope insertion site in adenovirus hexon. J. Virol..

[40] Villalta F., Lima M.F., Ruiz-Ruano A., Zhou L. (1992). Attachment of *Trypanosoma cruzi* to host cells: A monoclonal antibody recognizes a trypomastigote stage-specific epitope on the gp 83 required for parasite attachment. Biochem. Biophys. Res. Commun..

[41] Parks R.J. (2005). Adenovirus protein IX: A new look at an old protein. Mol. Ther..

[42] Meulenbroek R.A., Sargent K.L., Lunde J., Jasmin B.J., Parks R.J. (2004). Use of adenovirus protein IX (pIX) to display large polypeptides on the virion—Generation of fluorescent virus through the incorporation of pIX-GFP. Mol. Ther..

[43] Le L.P., Li J., Ternovoi V.V., Siegal G.P., Curiel D.T. (2005). Fluorescently tagged canine adenovirus via modification with protein IX-enhanced green fluorescent protein. J. Gen. Virol..

[44] Li J., Le L., Sibley D.A., Mathis J.M., Curiel D.T. (2005). Genetic incorporation of HSV-1 thymidine kinase into the adenovirus protein IX for functional display on the virion. Virology.

[45] Li J., Fatima A., Komarova S., Ugai H., Uprety P., Roth J.C., Wang M., Oster R.A., Curiel D.T., Matthews Q.L. (2010). Evaluation of adenovirus capsid labeling *vs.* transgene expression. Virol. J..

[46] Tang Y., Le L.P., Matthews Q.L., Han T., Wu H., Curiel D.T. (2008). Derivation of a triple mosaic adenovirus based on modification of the minor capsid protein IX. Virology.

[47] Gamble L.J., Borovjagin A.V., Matthews Q.L. (2010). Role of RGD-containing ligands in targeting cellular integrins: Applications for ovarian cancer virotherapy (review). Exp. Ther. Med..

[48] Gamble L.J., Ugai H., Wang M., Borovjagin A.V., Matthews Q.L. (2012). Therapeutic efficacy of an oncolytic adenovirus containing RGD ligand in minor capsid protein IX and Fiber, Δ24DoubleRGD, in an ovarian cancer model. J. Mol. Biochem..

[49] Gu L., Krendelchtchikova V., Krendelchtchikov A., Farrow A.L., Derdeyn C.A., Matthews Q.L. (2016). Adenoviral vectors elicit humoral immunity against variable loop 2 of clade C HIV-1 gp120 via “antigen capsid-incorporation” strategy. Virology.

[50] Pan A.A., McMahon-Pratt D. (1989). Amastigote and epimastigote stage-specific components of *Trypanosoma cruzi* characterized by using monoclonal antibodies. Purification and molecular characterization of an 83-kilodalton amastigote protein. J. Immunol..

[51] Low H.P., Santos M.A., Wizel B., Tarleton R.L. (1998). Amastigote surface proteins of *Trypanosoma cruzi* are targets for CD8^+^ CTL. J. Immunol..

[52] Lima M.F., Villalta F. (1989). *Trypanosoma cruzi* trypomastigote clones differentially express a parasite cell adhesion molecule. Mol. Biochem. Parasitol..

[53] Silveira E.L., Claser C., Haolla F.A., Zanella L.G., Rodrigues M.M. (2008). Novel protective antigens expressed by *Trypanosoma cruzi* amastigotes provide immunity to mice highly susceptible to Chagas’ disease. Clin. Vaccine Immunol..

[54] Barbosa R.P., Filho B.G., Dos Santos L.I., Junior P.A., Marques P.E., Pereira R.V., Cara D.C., Bruna-Romero O., Rodrigues M.M., Gazzinelli R.T. (2013). Vaccination using recombinants influenza and adenoviruses encoding amastigote surface protein-2 are highly effective on protection against *Trypanosoma cruzi* infection. PLoS ONE.

[55] Dmitriev I.P., Kashentseva E.A., Curiel D.T. (2002). Engineering of adenovirus vectors containing heterologous peptide sequences in the C terminus of capsid protein IX. J. Virol..

[56] Gu L., Li Z.C., Krendelchtchikov A., Krendelchtchikova V., Wu H., Matthews Q.L. (2013). Using multivalent adenoviral vectors for HIV vaccination. PLoS ONE.

[57] Sargent K.L., Meulenbroek R.A., Parks R.J. (2004). Activation of adenoviral gene expression by protein IX is not required for efficient virus replication. J. Virol..

[58] Miyahira Y., Takashima Y., Kobayashi S., Matsumoto Y., Takeuchi T., Ohyanagi-Hara M., Yoshida A., Ohwada A., Akiba H., Yagita H. (2005). Immune responses against a single CD8^+^-T-cell epitope induced by virus vector vaccination can successfully control *Trypanosoma cruzi* infection. Infect. Immun..

[59] Parodi C., Padilla A.M., Basombrio M.A. (2009). Protective immunity against trypanosoma cruzi. Mem. Inst. Oswaldo Cruz.

[60] Dos Santos Virgilio F., Pontes C., Dominguez M.R., Ersching J., Rodrigues M.M., Vasconcelos J.R. (2014). Cd8(+) T cell-mediated immunity during *Trypanosoma cruzi* infection: A path for vaccine development?. Mediat. Inflamm..

[61] Rux J.J., Burnett R.M. (2004). Adenovirus structure. Hum. Gene Ther..

[62] Coughlan L., Bradshaw A.C., Parker A.L., Robinson H., White K., Custers J., Goudsmit J., van Roijen N., Barouch D.H., Nicklin S.A. (2012). Ad5:Ad48 hexon hypervariable region substitutions lead to toxicity and increased inflammatory responses following intravenous delivery. Mol. Ther..

[63] Barouch D.H., Kik S.V., Weverling G.J., Dilan R., King S.L., Maxfield L.F., Clark S., Ng’ang’a D., Brandariz K.L., Abbink P. (2011). International seroepidemiology of adenovirus serotypes 5, 26, 35, and 48 in pediatric and adult populations. Vaccine.

[64] Parker A.L., Waddington S.N., Buckley S.M., Custers J., Havenga M.J., van Rooijen N., Goudsmit J., McVey J.H., Nicklin S.A., Baker A.H. (2009). Effect of neutralizing sera on factor X-mediated adenovirus serotype 5 gene transfer. J. Virol..

[65] Waddington S.N., McVey J.H., Bhella D., Parker A.L., Barker K., Atoda H., Pink R., Buckley S.M., Greig J.A., Denby L. (2008). Adenovirus serotype 5 hexon mediates liver gene transfer. Cell.

[66] Farrow A.L. (2015). Sequencing and PCR analysis of viral genomes.

[67] Matthews Q.L., Sibley D.A., Wu H., Li J., Stoff-Khalili M.A., Waehler R., Mathis J.M., Curiel D.T. (2006). Genetic incorporation of a herpes simplex virus type 1 thymidine kinase and firefly luciferase fusion into the adenovirus protein S for functional display on the virion. Mol. Imaging.

[68] Matthews Q.L., Yang P., Wu Q., Belousova N., Rivera A.A., Stoff-Khalili M.A., Waehler R., Hsu H.C., Li Z., Li J. (2008). Optimization of capsid-incorporated antigens for a novel adenovirus vaccine approach. Virol. J..

